# Validation of the AX3 triaxial accelerometer in older functionally impaired people

**DOI:** 10.1007/s40520-016-0604-8

**Published:** 2016-07-19

**Authors:** Clare L. Clarke, Judith Taylor, Linda J. Crighton, James A. Goodbrand, Marion E. T. McMurdo, Miles D. Witham

**Affiliations:** Ninewells Hospital and Medical School, School of Medicine, University of Dundee, Dundee, UK

**Keywords:** Physical activity, Older adults, Ageing, Accelerometry, Public health

## Abstract

**Background:**

Studying physical activity (PA) trends in older populations and potential interventions for increasing PA is important, as PA is a factor in many age-related health outcomes such as chronic disease, premature mortality, physical function and injuries from falls. Objective measures of PA provide valuable information regarding the functional impact that ageing and chronic disease states may have on a patient’s life.

**Aims:**

The purpose of this study was to test the validity of the AX3 PA monitor in an older population and to investigate whether the AX3 is a valid measure of distinct types or levels of activity in older people with a spectrum of mobility.

**Methods:**

Validity of the AX3 PA monitor was tested using the RT3 as a means of cross-validating the AX3. Study participants wore both the AX3 and the RT3 accelerometers, positioned on their non-dominant side, whilst completing a series of standardised everyday activities.

**Results:**

Although overall correlation was high (*r* > 0.8) between the RT3 and lower-limb-mounted AX3 counts, the correlation between the two devices was much stronger for walking activity than for any of the non-walking activities.

**Discussion:**

Activity counts at all lower limb positions for the AX3 and RT3 were highly correlated. Correlation between wrist-mounted AX3 counts and lower limb AX3 counts was only moderate, and worsened when walking aids were in use.

**Conclusions:**

The results of this study indicate that the AX3 monitor is a valid tool, which might be used to objectively measure walking activity in older functionally impaired adults, a welcome finding for this under-researched area.

## Introduction

Researching physical activity (PA) trends in older populations and potential interventions for increasing PA is important. PA is a factor in many age-related health outcomes such as chronic disease, premature mortality, physical function and injuries from falls [1]. It is also highly modifiable, which is important given the fact that people aged 65 and over are the most sedentary of any age group [1]. It is not surprising that the use of objective methods of measuring PA has become more commonplace in public health research over the last decade [2]. There is a clear need for methods of accurately and objectively capturing how much PA older people do. In order to be considered accurate, the method should be objective and valid for the observed population, measuring without bias what people actually do, whilst accounting for factors such as slower walking speeds [3] and dependence on walking aids. Objective measures of PA can provide valuable information regarding the functional impact that ageing and chronic disease states may have on a patient’s life and could aid in the monitoring and evaluation of interventions designed to improve symptoms [4].

Triaxial accelerometers offer advantages in that they are objective and so free of the biases inherent in asking the patient to recall how much they have done (i.e. self-report). They also have the potential to produce a richer data set, with detailed information on how activity changes from minute to minute. Triaxial accelerometers are worn on the body typically over a 7-day period and measure acceleration along three orthogonal axes. The captured data can be used to assess multiple dimensions of PA [5]. The devices are becoming smaller, lighter and therefore less obtrusive to the wearer. The RT3 accelerometer has been used to measure PA in numerous studies in the past decade and must be clipped over the hip at the waistband. However, the RT3 devices use old technology and are no longer commercially available. Technological advances in PA monitors have progressed significantly in recent years, and so there is a need to determine the validity of newer models as objective measures of PA, in a variety of populations. The AX3 has not been validated for use in older adults and so we aimed to evaluate the Axivity AX3 PA monitor for use in this subgroup.

The AX3 is substantially smaller (matchbox size), is lighter (weight 16 vs 64 g for the RT3) and can be worn on the wrist, ankle or various other sites such as the thigh. It is also waterproof to 1.5 m and so is perhaps more tolerant to any need or desire to remove the device, which may ensure more complete data collection. Previous validity tests of the AX3 accelerometer have used a shaker table [6] and younger people carrying out physical training exercises [7]. The purpose of this study is to test the validity of the AX3 in an older population, in a similar manner to our previous validation of the RT3 device [8], but additionally using the RT3 as a means of cross-validating the AX3.

## Methods

### Study participants

We recruited participants aged over 65 from inpatient and outpatient Medicine for the Elderly Dundee services and from a panel of local older volunteers. All participants gave written informed consent. Ethical approval was granted by the Tayside Local Research Ethics Committee (13/ES/0120) and the study conformed to the principles of the Declaration of Helsinki. Participants were all independently mobile without human assistance and were recruited with the following characteristics: no activity limitations and no walking aids; activity limitation but no walking aid; activity limitation and use of one walking stick; activity limitation and use of Zimmer frame; activity limitation and use of triwheel walker.

### Accelerometers

**Fig. 1:**
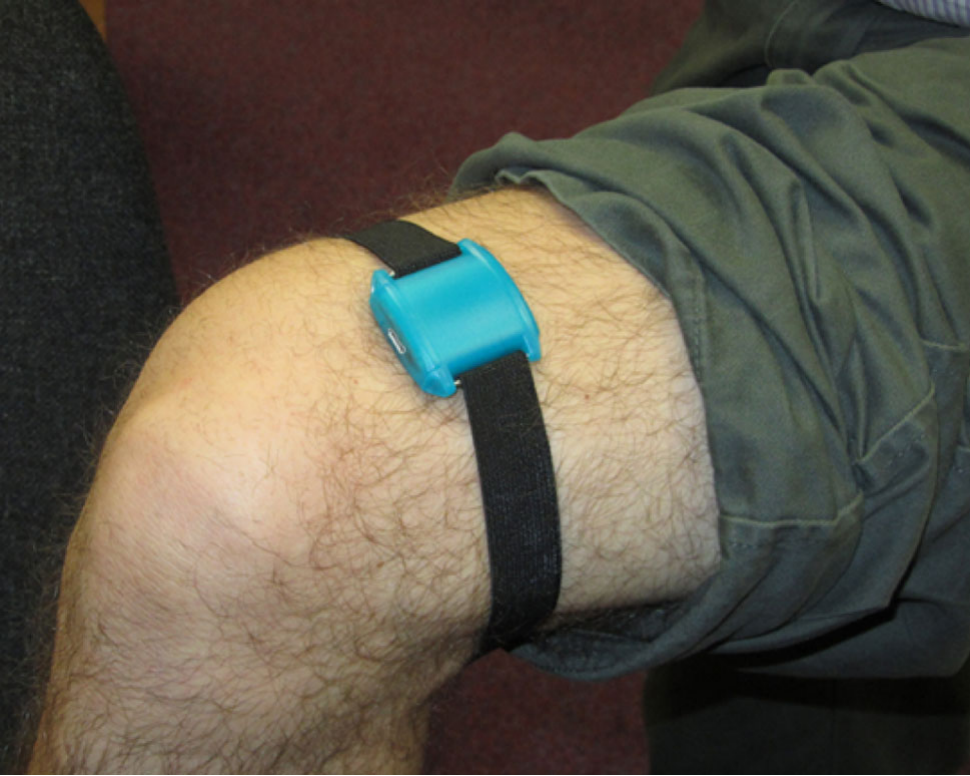
AX3 mounted on thigh

The AX3 accelerometer is a small lightweight triaxial accelerometer, weighing 16 g. The AX3 accelerometer is usually used with an attachment band for use on the wrist; for the purposes of this study, the AX3 was mounted either on elasticated bands (for use on ankle, thigh or wrist) or on a belt clip adjacent to the RT3 accelerometer on the non-dominant side (see Fig. 1). For each participant, a total of four AX3 and one RT3 accelerometers were used.

### Study visits and outcomes

The study schedule involved a single study visit. Baseline information was collected on current activity levels, walking aids, age, sex, height, weight and comorbid disease. Study participants wore both the AX3 and the RT3 accelerometers, positioned on their non-dominant side, whilst completing a series of standardised activities that have been reported in detail previously [8]. Each participant completed the activities in the same order, with each activity lasting 6 min: standing activity, walking, seated rest, seated activity, lying supine and stair-climbing activity (optional, only for those participants able to climb stairs).

For the standing and seated activity, participants were asked to move rings over cones placed on a table at set positions based on the participant’s demispan, as previously described [8]. The 6-min walk was performed in a corridor 15 m long, with standardised encouragement every 30 s. The distance travelled and number of rests were recorded. During the seated rest, participants were allowed to talk and make gestures. For the 6 min of lying supine, participants were encouraged to stay still. Participants completing the stair climb were asked to climb up three steps to a platform and down twice, and then given a rest until the end of each minute. The researcher recorded as secondary outcomes the distance travelled and number of rests in the 6-min walk, the number of cones moved in the standing and sitting tasks and the number of times up and down the steps in the stair-climbing task.

At the start of each 6-min period, participants were asked to clap three times at the same time as the RT3 event marker button was pushed by the researcher. This provided a record on the data files of when each activity started. RT3 recorders were used in mode 3 (1-min epochs recorded); AX3 recorders were used recording at 100 Hz.

### Analyses

The sum of vector magnitude counts per minute was obtained from the RT3 data file. The start of each minute epoch was denoted by a time stamp; times for the RT3 and AX3 time stamps were derived from a single computer, used to initiate all the recorders. For the RT3, the first and last minute of each activity was discarded, as activities are likely to have started and stopped during the minute and hence less than a whole minute of activity would have been recorded. Five values (1 min each) per activity were therefore derived for each participant from the RT3.

For AX3 data, the start time for data reduction was taken as the start of the first whole minute of data, using the time stored on the RT3 to ensure that the time periods were synchronised. The presence of handclap spiked during the minute prior to this point on the AX3 data served as a check that the data epochs were synchronised correctly. Vector magnitude counts were calculated for each 1/100th of a second from the three orthogonal AX3 data channels using OMGUI software (GITHub, University of Newcastle); the signal vector magnitude function was used for these calculations using a filter bandpass of 0.5–20 Hz. In addition, the angle between the *x* acceleration vector (expressed as a fraction of *g*) and the *y* and *z* vectors was calculated. Vectors were then summed into five 1-min epochs after the start time, and the mean acceleration angle relative to the *y*/*z* plane was also calculated for each 1-min epoch.

All statistical analyses were performed using SPSS version 21 (IBM, New York, USA). A two-sided *p* value of <0.05 was taken as significant for all analyses. Correlation coefficients were calculated using Pearson’s test, comparing each individual minute of activity between accelerometers. For calculating the optimum cut point between walking and non-walking activity, Youden’s index (sensitivity + specificity-1) was calculated for each potential cut-off; the highest index value was taken as the optimum cut-off point.

## Results

### Study population

A total of 23 participants with usable data were included. Baseline details for the participant groups are given in Table 1. Participants with no activity limitations were, as expected, much fitter than those needing to use walking aids; those using walking aids had a higher burden of comorbid disease and took more medications.

**Table 1:** Baseline details of participant groups

Group	A (no AL, no walking aids)	B (AL, no walking aids)	C (AL, walking stick)	D (AL, walking frame)	E (AL, triwheel walker)
N	6	4	5	4	4
Female	4/6	2/4	4/5	3/4	2/5
Mean age (SD)	73 (6)	81 (8)	77 (11)	81 (10)	82 (10)
Mean number of comorbidities (SD)	0.8 (1.2)	1.4 (1.1)	2.4 (1.7)	1.5 (1.3)	2.6 (1.8)
Mean number of medications (SD)	1 (0.8)	5 (3)	7 (4)	11 (2)	10 (5)
Mean 6-min standing activity—no. of rings (SD)	167 (42)	120 (11)	113 (32)	78 (50)	86 (31)
Mean 6MWT distance in m (SD)	410 (40)	324 (74)	200 (21)	66 (31)	99 (55)
Mean 6-min sitting activity—no. of rings (SD)	154 (36)	127 (23)	124 (44)	89 (57)	108 (19)
Mean 6-min step-climbing activity—time taken for each group of steps in secs (SD)	13 (3)	18 (3)	33 (16)	39 (–)*	77 (11)

*AL* activity limitation

* Only one of four participants completed step-climbing activity

There were moderate bivariate correlations for AX3 counts between wrist and ankle, hip and thigh (0.69, 0.73 and 0.70, respectively; all *p* < 0.001); correlations between AX3 lower limb positions were high (ankle vs hip *r* = 0.98; ankle vs thigh *r* = 0.97; hip vs thigh *r* = 0.95; all *p* < 0.001).

Table 2 shows the correlation between AX3 counts in different positions and the hip-mounted RT3 counts. Although overall correlation was high (*r* > 0.8) between the RT3 and lower-limb-mounted AX3 counts, the correlation between the two devices was much stronger for walking activity than for any of the non-walking activities. Correlations between the RT3 and the wrist-mounted AX3 were lower. Table 2 also shows equations derived from the correlation plots to estimate AX3 counts in each tested position from hip-mounted RT3 counts.

**Table 2:** Correlation between RT3 counts and AX3 counts (all participants)

	Standing task	Walking	Rest	Sitting task	Lying	Steps	All activities combined	Equation
AX3 Wrist	0.33 (<0.001)	0.87 (<0.001)	0.30 (0.001)	0.01 (0.90)	0.26 (0.004)	0.25 (0.02)	0.69 (<0.001)	AX3 = (RT3 × 0.606) + 175
AX3 Ankle	0.22 (0.02)	0.90 (<0.001)	−0.01 (0.91)	−0.02 (0.87)	0.20 (0.03)	0.34 (0.001)	0.88 (<0.001)	AX3 = (RT3 × 2.161) – 26
AX3 Hip	0.36 (<0.001)	0.91 (<0.001)	0.26 (0.006)	−0.04 (0.70)	0.24 (0.01)	0.58 (<0.001)	0.89 (<0.001)	AX3 = (RT3 × 0.845) + 19
AX3 Thigh	0.27 (0.004)	0.59 (<0.001)	−0.05 (0.56)	0.06 (0.52)	0.13 (0.18)	0.04 (0.71)	0.88 (<0.001)	AX3 = (RT3 × 1.294) − 2

*p* values are given in brackets

Marked differences in the strength of correlation between the RT3 and AX3 were noted for different types of walking aid use. Correlations at all sites were high for those not using walking aids; correlations were lower for those using a stick or wheeled walker and were poor for those using a Zimmer frame; correlations were also poor between the RT3 and the wrist-mounted AX3 for those using a wheeled walker. Details of all correlations are given in Table 3.

**Table 3:** Correlations (for all activities) subdivided by type of walking aid

RT3 versus:	Fit, no aids	Impaired, no aids	Impaired, walking stick	Impaired, Zimmer frame	Impaired, triwheel walker
Wrist	0.76 (<0.001)	0.68 (<0.001)	0.53 (<0.001)	0.42 (<0.001)	0.14 (0.14)
Ankle	0.92 (<0.001)	0.98 (<0.001)	0.79 (<0.001)	0.29 (0.003)	0.70 (<0.001)
Hip	0.92 (<0.001)	0.98 (<0.001)	0.77 (<0.001)	0.36 (<0.001)	0.86 (<0.001)
Thigh	0.91 (<0.001)	0.98 (<0.001)	0.78 (<0.001)	0.30 (<0.001)	0.77 (<0.001)

Table 4 shows the mean counts for different activities for each accelerometer position, along with the optimum cut point to distinguish walking from other activities for each accelerometer position. Table 5 shows mean angles and cut-offs for each AX3 accelerometer position.

**Table 4:** Mean counts for each activity (range) [95 % CI]

	Standing task	Walking	Rest	Sitting task	Lying	Steps	Optimal cut-off* (counts/min)	Youden’s index
RT3	83 (0–330) [67–99]	681 (31–2174) [594–768]	61 (0–1047) [37–86]	120 (0–1486) [78–161]	15 (0–247) [8–23]	204 (40–1085) [178–229]	420	0.826
Wrist	372 (50–1098) [331–414]	569 (82–1410) [494–644]	106 (7–434) [90–122]	337 (32–1049)[294–380]	39 (6–225)[33–46]	339 (53–678) [318–361]	600	0.527
Ankle	33 (14–67) [31–36]	1802 (54–4597) [1605–2001]	32 (2–201) [26–38]	28 (8–62) [26–30]	18 (2–64) [16–20]	458 (85–1000) [432–485]	800	0.969
Hip	72 (30–127) [68–76]	697 (43–2004) [613–780]	31 (6–68) [28–33]	48 (22–87) [45–50]	23 (8–91) [21–25]	242 (67–572) [228–256]	420	0.916
Thigh	43 (18–93) [41–46]	1046 (31–2534) [915–1176]	32 (9–142) [28–36]	34 (18–70) [32–36]	21 (9–64) [19–22]	340 (71–737) [321–359]	500	0.933

* Cut-off to differentiate walking from other activities

**Table 5:** Mean angle of vectors from AX3 for each activity (range in degrees) [95 % CI]

	Standing task	Walking	Sitting Rest	Sitting task	Lying	Steps	For lying versus not lying		For lying/sitting versus standing/walking/steps	
							Optimal cut-off angle (°)	Youden’s index	Optimal cut-off angle (°)	Youden’s index
Wrist	47 (15–78) [44–50]	62 (5–80) [58–66]	24 (3–82) [20–28]	24 (7–58) [21–26]	21 (1–66) [17–25]	37 (11–76) [33–40]	12	0.418	24	0.512
Ankle	77 (60–86) [76–77]	68 (56–80) [67–69]	69 (34–86) [66–71]	71 (53–87) [69–73]	4 (0–41) [3–5]	72 (43–84) [71–74]	41	0.998	61	0.442
Hip	57 (9–77) [53–60]	62 (19–80) [58–65]	49 (5–80) [44–53]	56 (5–79) [52–61]	15 (0–44) [12–17]	63 (13–85) [58–67]	46	0.787	51	0.367
Thigh	76 (34–88) [74–78]	66 (26–79) [64–67]	19 (1–82) [14–24]	22 (2–86) [18–27]	10 (0–21) [8–11]	68 (4–81) [65–72]	32	0.611	38	0.908

## Discussion

We found that although activity counts at all lower limb positions for the AX3 and RT3 were highly correlated, correlation between wrist-mounted AX3 counts and lower limb AX3 counts was only moderate, and was worse when walking aids were in use. This finding is in line with other studies that reported a higher correlation with systems placed on the lower limbs rather than upper limbs, especially when focusing on relevant functional tasks demanding of the lower limb [9]. Correlations at all sites were high for those not using walking aids; correlations were lower for those using a stick or wheeled walker and were poor for those using a Zimmer frame. Correlations were also poor between the RT3 and the wrist-mounted AX3 for those using a wheeled walker.

We found that it was possible to derive equations to relate RT3 counts to AX3 counts, which may prove useful in comparing activity levels in different older populations across studies. We found that the AX3 monitor was able to accurately distinguish walking activity from non-walking activities when used in any of the three lower limb positions, that only an ankle-mounted AX3 accurately discriminated lying from non-lying activity and that only a thigh-mounted AX3 accurately discriminated sedentary from non-sedentary activity. The wrist-mounted AX3 accelerometer was not able to accurately discriminate between any of these groups of activity in older functionally impaired people.

There is still considerable debate over both the choice of accelerometer to use and the ideal location of the accelerometer(s) for different applications, as the acceleration signal recorded from the body depends upon the design and location of the sensing device and the activity being performed. For example, using an accelerometer on the upper limb carries higher face validity for measuring upper limb activities, but would not be expected to accurately reflect lower limb activity. Attempting to use waist-worn accelerometers to detect sedentary behaviour has been shown to be problematic. Waist-worn accelerometers have been reported as being incapable of distinguishing between different postures, and soft tissue motion at the waist can induce significant errors in belt worn devices that can lead to periods of standing being misclassified as sedentary [10]. Data from the hip have been shown to be the best single location to put an accelerometer to distinguish between a range of activities, and our results would go someway to support this [9]. In May 2013, the company *Stayhealthy, Inc., Monrovia, CA,* introduced the RT6 which has superseded the RT3. The RT6 has a triaxial accelerometer and three-axis gyroscopes. This configuration is reported to overcome the problems associated with exercise where the waist is stationary. This monitor was not available at the time these data were collected.

The ability to provide accurate information would depend on a user’s activity and also the context in which the device is used. This may be particularly important in measuring activity in older functionally impaired people—whilst in young, fit people, a given activity (e.g. walking, sport activity) will tend to generate movements at the arms, legs and trunk, this is unlikely to be the case for older, impaired people. Individuals who use a Zimmer frame or walker lose the natural coordination of limbs during walking; arms holding a walking aid will not move when a step is taken; hips may not move much when a shuffling step is made. Hence, a disconnection between activity measurements at different parts of the body is more likely in older people, making a proxy choice of measuring site less appropriate.

UK Biobank is a prospective study of 500 000 UK participants recruited in middle age during 2006–2010 [11]. Extensive data were collected at baseline from all participants on their lifestyle, environment, personal and family medical history. The UK Biobank favoured the AX3 device over the others as it provides raw un-filtered actigraphy data, is a fully well-documented open-source product, is postal friendly and is value for money [12]. Khan et al. [13] used the AX3 monitor to validate its use through a range of everyday activities. The device was placed on the lower part of the spine and was able to predict all physical activities with the accuracy of more than 80 %. Lying, walking, sitting, standing and cycling activities were predicted with the accuracy of more than 95 %. Walking activity was predicted 100 % the ‘J48 classifier’ algorithm. Further research was recommended investigating its validity when located on different body parts.

The results of this study indicate that the AX3 monitor is a valid tool which can be used to objectively measure walking activity in older functionally impaired adults, which is a welcome finding for this under-researched population [13, 14]. Cross-validation with the RT3 monitor in our previous work strengthens our results and allows comparison across studies using these different measures.

Our study has a number of limitations, however, in that our sample size was small and the use of walking aids appears to interfere with measurement. Our focus on people with walking aids means that our ability to draw conclusions about fitter older people is limited. Larger samples may be required to reveal differences between other non-walking activities. We used simple cut-offs for both activity count and acceleration angle to discriminate between activities. It is possible that more sophisticated signal analysis will be able to better discriminate between activities, especially given the high temporal resolution (100 Hz) of AX3 data. Such approaches like that of Khan et al. [13] referred to earlier might allow sedentary versus non-sedentary activity to be discriminated at sites including the wrist, which was not possible using our analytical approach.

The measurement of finer-skilled PA needs further investigation. These activities, which have more subtle differences in acceleration, such as working in a sitting posture, may require different approaches (e.g. using multiple accelerometers) to detect. Further work should therefore focus on identifying which combination of accelerometer position provides the best accuracy for these finer-skilled activities. Additionally, the accuracy of such classifiers should be assessed under free-living conditions, as measuring PA in the laboratory does not necessarily translate to performance in the real world [4, 9].

## Conclusion

The AX3 is a valid tool that might be used to objectively measure walking activity in older functionally impaired adults. Caution must be taken, however, as the use of walking aids may interfere with measurement. There is a real need to assess the validity of existing accelerometers to record accurately the more intricate upper limb activities occurring in everyday life, in addition to slow walking in older adults (*especially those who use walking aids*) as this information is important in the design of interventions designed to improve PA levels in this population.
